# The role of infectious agents in urogenital cancers

**DOI:** 10.1186/1750-9378-7-35

**Published:** 2012-12-03

**Authors:** Kenneth Alibek, Nargis Karatayeva, Ildar Bekniyazov

**Affiliations:** 1 Nazarbayev University, 53 Kabanbay Batyr Avenue, Astana 010000, Kazakhstan; 2 Republican Scientific Center for Emergency Care, 3 Kerey and Zhanibek Khan Street, Astana 010000, Kazakhstan

**Keywords:** Urogenital cancers, Ovarian cancer, Cervical cancer, Bladder cancer, Kidney cancer, Chlamydia trachomatis, Neisseria gonorrhea, Mycoplasma genitalium, Schistosoma haematobium, HPV, Epstein-Barr, CMV, Human polyomaviruses

## Abstract

Since the late 1990s, infectious agents have been thought to play a role in the pathogenesis of approximately 15% of cancers. It is now widely accepted that infection of stomach tissue with the bacteria *Helicobacter pylori* is an important cause of stomach adenocarcinoma. In addition, oncogenic viruses, such as papilloma viruses, herpes viruses, and hepadnaviruses are strongly associated with increased risk of cervical cancer, lymphomas, liver cancer, amongst others. However, in the scientific community the percentage of cancers caused by pathogens is believed to be far higher than 15%. A significant volume of data collected to date show an association between infectious agents and urogenital cancers. These agents include *Chlamydia trachomatis*, *Neisseria gonorrhoea*, *Mycoplasma genitalium* and certain viruses that have been implicated in ovarian cancer. Other pathogens include the hepatitis C and Epstein-Barr viruses, which are potentially involved in kidney cancer. In addition, infections with *Schistosoma haematobium*, the human papillomavirus, and human polyomaviruses are strongly associated with an increased risk of urinary bladder cancer. This article reviews publications available to date on the role of infectious agents in urogenital cancers. A greater understanding of the role of such agents could aid the identification of novel methods of urogenital cancer treatment.

## 

Urogenital tract cancers including ovarian, cervical, kidney and bladder carcinomas are among the most common and lethal cancers worldwide. Numerous factors could be involved in urogenital cancer development, including genetic predisposition, lifestyle, and infectious agents. Approximately 15% of cancers worldwide are caused by microorganisms [1], but growing evidence indicates this number might be much higher. This article reviews literature available to date on the role of microorganisms in urogenital tract cancers. A greater understanding of the function of such microorganisms in promoting carcinogenesis is required to aid identification of novel strategies for cancer treatment.

## Ovarian cancer

Ovarian carcinoma is a common human tumor of the genital tract and one of the most lethal cancers worldwide [2]. In 2008, ovarian carcinoma was the 2nd most lethal gynecological cancer [3]. The molecular events occurring during early stages of ovarian carcinogenesis are not well understood due to the absence of symptoms in patients and the site of origins of neoplastic lesions. Most patients are diagnosed with ovarian cancer when the tumor is at an advanced stage and has metastasized to the peritoneum. The prognosis of ovarian cancer is therefore poor. Moreover, ovarian cancers rapidly develop resistance to cytotoxic drugs [4]. Piek et al. [5] list three possible routes of ovarian carcinogenesis: ovarian surface epithelial tissue, embryologic Mullerian ducts, and the inner surface epithelium of the Fallopian tube.

Most aggressive ovarian carcinomas arise from ovarian surface epithelium (OSE) cells and account for about 60% of all ovarian carcinomas. Non-epithelial ovarian tumors arising from oocytes and sex cord stromal cells usually remain benign. Thus, ovarian epithelial tumors receive greater attention, and multiple studies are currently underway to determine the origins of neoplastic lesions in the OSE and understand the early molecular events of the disease. Two broadly accepted theories of OSE carcinogenesis are incessant ovulation and gonadotropin. The incessant ovulation theory suggests the epithelium may be more apt to mutate with each ovulation and failure to break the cycle, as in pregnancy, increases the chance of mutation. The gonadotropin theory suggests elevated gonadotropin levels trigger proliferation of the ovarian epithelium, with subsequent malignant transformation. Epidemiological data available to date do not provide sufficient evidence for either theory [6,7].

In a third theory, Ness and Cottreau [8] suggested that inflammatory-like processes, occurring during ovulation and mediated by gonadotropins, underlie higher cancer risks associated with ovulation and gonadotropin stimulation. This inflammation theory is supported by several epidemiological studies focused on mechanisms that did not affect hormone levels and ovulation, including effects of asbestos and talc exposure, prophylactic hysterectomy and tubal ligation, pelvic inflammatory disease and endometriosis [9-17]. It was previously established that chronic inflammation, a response of the organism to pathogens or irritants, could lead to neoplastic transformation of inflamed tissues due to host defense mechanisms and their products, which were thought to have antimicrobial properties [18]. During inflammation, mutagenic substances such as reactive oxygen and nitrogen species are released by neutrophils and macrophages and play a crucial role in promoting carcinogenesis. These species can directly damage DNA by oxidation and nitration, and modify cancer-related proteins [19]. In combination with elevated levels of cell proliferation for repair of damaged tissue, these changes create favorable conditions for increased transformation.

Apoptosis is another critical cellular mechanism that eliminates damaged or unwanted cells by directing them to undergo programmed cell death. It is also a vital process that contributes significantly to the protection from tumor formation. Thus inhibition of apoptosis increases the risk of malignancy, and it was found that cells infected with *Chlamydia trachomatis* (*C*.*trachomatis*) and human papillomavirus (HPV) are resistant to apoptosis [20,21].

Various microorganisms involved in sexually transmitted diseases, including *C*.*trachomatis and Mycoplasma genitalium* (*M*.*genitalium*), have been associated with a higher risk for ovarian cancer [22,23]. These pathogens cause chronic and asymptomatic infection, increasing the risk of neoplastic transformation of the OSE. These pathogens are also associated with pelvic inflammatory disease (PID) and tubal infertility factor (TIF), suggesting they can traverse from the lower to upper genital tract infecting and causing persistent inflammation in the Fallopian tubes and ovaries. These associations indicate that more attention should be paid to the role of these pathogens in ovarian cancer.

*C*.*trachomatis* is the most prevalent infection of the genital tract in women worldwide, and the disease is usually asymptomatic [24]. It is possible to suggest that cells infected by chlamydia have a much higher risk of neoplastic transformation because the pathogen is able to cause persistent inflammation and hence tissue damage, and has an antiapoptotic activity on infected cells [21]. Chlamydia is an obligatory intracellular pathogen and can replicate only inside a cytoplasmic vacuole (phagosome) of the host cell. It is able to cause chronic asymptomatic infection due to the fact that it can evade the host immune response by preventing the fusion of the phagosome with a lysosome by an unknown mechanism [25], by inhibiting expression of the host cell’s major histocompatibility complex antigen [26-29], and by blocking pathways to apoptosis. In the study by Greene et al. [16], serovars of *C*. *trachomatis* and *C*.*psittaci* were analyzed and inhibition of apoptosis of infected cells in cultures was observed. Moreover, the authors observed that DNA synthesis was unaffected in infected cells and infected cells could undergo mitosis at any stage of infection which also increases the risk of malignant transformation of infected cells. The mechanisms and key players involved in blocking apoptosis in infected cells require further investigation. Some studies [30,31] suggest that chlamydia heat shock protein (a major cause of persistent inflammation) might have antiapoptotic activity. Serum antibodies to this protein were elevated in women with TFI [32]. Cooper et al. [33] observed that chlamydia also has a cytotoxic effect on cells promoting loss of microvilli and cell junctions, likely by producing a toxin (homologous to large clostridial cytotoxin) [34]. Loss of cell adhesion resulting from the loss of cell junctions induces selection of preneoplastic cells. It remains to be determined whether other bacterial toxins or proteins that can affect the host cell’s DNA directly.

*M*.*genitalium* is well associated with PID and TFI, and could also cause chronic asymptomatic infection in the upper genital tract. M.genitalium might increase the risk of neoplastic transformation of tubal and ovarian tissue. However, Idahl et al. [23] did not find an association between *M*.*genitalium* antibodies and ovarian tumors. Nonetheless, prolonged infection could cause morphological changes in mammalian host cells. A study by Baczynska et al. [35], demonstrated that *Mycoplasma hominis* (*M*.*hominis*) and *M*.*genitalium* caused swelling of the tubal epithelial cilia through production of a toxin or metabolism products – hydrogen peroxide and superoxide radicals. This is consistent with finding of recent studies suggesting some serous ovarian carcinomas (the most aggressive ovarian carcinoma) can originate from the Fallopian tube epithelium [5,36]. Another study by Namiki et al. [37] demonstrated that prolonged infection of benign human prostate cells in culture with *M*.*genitalium* and *Mycoplasma hyorhinis* caused malignant transformation through an unknown mechanism.

*Neisseria gonorrhea* (*N*.*gonorroea*) is a facultative intracellular pathogen that attaches to the apical surface of the genital tract epithelium. *Neisseria gonorrhea* has not been associated with ovarian cancer so far. However, the attachment of the pathogen to the epithelium of the genital tract provokes various cell signaling events and alters expression of many cellular proteins, which might predispose them to malignant transformation. *N*.*gonorrhoea* alters cell cycle progression by reducing expression of cyclin B and arresting cells in the G1 phase of the cell cycle [38]. Löfmark et al. [39] investigated Me-180 (cervical carcinoma cell line) and HEC-1-B (endometrial adenocarcinoma cell line) cells infected with *N*.*gonorrhoea* and examined their levels of amphiregulin expression. Amphiregulin is an epidermal growth factor receptor ligand, expressed in the G1 stage that promotes cellular growth and cell cycle progression. The role of amphiregulin in various cancers has been extensively investigated. Amphiregulin appears to contribute to tumorigenesis by promoting independent production of growth signals, limitless proliferation, tissue invasion and metastasis, angiogenesis, and resistance to apoptosis (reviewed in [40]). Amphiregulin was expressed at higher rates following infection in Me-180 and HEC-1-B cell lines. However, the effect of amphiregulin on OSE cells and the ability of *N*.*gonorrhoea* to cause their malignant transformation require elucidation.

A recent study by Shanmughapriya, et al. [41], showed HPV and cytomegalovirus (CMV) DNA were present in fresh ovarian carcinoma tissue samples in 40% and 50% of samples, respectively. The samples were collected from 39 women in total. The authors assumed that the HPV infection could arise from HPV-coated sperm that contact upper genital tract tissues. Ovarian CMV infections are quite rare and the authors therefore suggest that activation of the virus occurs during immunocompromised states that may occur following chemotherapy, radiotherapy, and metastatic disease. It was suggested that human cytomegalovirus is able to induce oncomodulation in infected cells, in other words the virus creates a more malignant phenotype of tumor cells by the action of its regulatory proteins and non-coding RNA which will affect their proliferation, invasion of other tissues, survival and other cellular properties [42]. However further investigation is necessary to determine whether ovaries are the primary site of CMV infection.

## Cervical cancer

Cervical cancer arises in the cervix uteri and is the most prevalent gynaecological malignancy worldwide [2]. HPV infection with high-risk HPV (16 and 18) is one of the most common causes of cervical cancer [43], other pathogens like *C*.*trachomatis*[44] and Epstein-Barr virus [45-47] were also found to be implicated in cervical carcinoma acting either independently or as co-factors in predisposing cervical epithelial cells to malignancy. Other factors that increase the risk of developing cervical carcinoma are genetic predisposition, smoking, weakened immunity, life style.

HPV is a small DNA virus that infects various epithelial tissues, and two types of the virus, HPV 16 and 18, are responsible for 70% of cervical carcinomas [43]. The virus inserts itself into human DNA, producing proteins that cause neoplastic transformation of the cervical epithelial cells. The key players involved in this process are early proteins 6 and 7 (E6 and E7), which are produced by HPV 16 and HPV 18 respectively. The viral DNA does not disrupt any human genes; instead, protein products of the viral genes - E6 and E7 proteins bind to the human tumor-suppressor proteins p53 and pRb, respectively, and inactivate them by promoting protein degradation (reviewed in [31]). Inactivation of these tumor-suppressors eventually leads to accumulation of mutations and onset of cancer. However, transient HPV infection is not sufficient to cause cervical neoplasia; it was previously observed that the infection could be cleared in most women [48,49]. The infection has to be persistent to cause transformation.

As some studies have shown, *C*.*trachomatis* increases the risk of HPV infection becoming persistent by increasing HPV’s access to the basal epithelium due to microabrasions of the cervical epithelium or changing its characteristics, or by preventing efficient clearance of the viral infection [50,51]. Also a study by Lehtinen et al. [44] demonstrated that C.trachomatis infection was an independent risk factor for cervical neoplasia. This could be because the pathogen blocks pathways to apoptosis in infected cells while promoting their entry to the S phase, causing chronic inflammation. Other sexually transmitted diseases could play a similar role to *C*.*trachomatis*, leading to persistence of HPV infection. The mechanisms involved in this process still need to be determined.

The correlation between Epstein-Barr virus (EBV) infection and uterine cervical cancer has been reported in several studies [45-47]. The role of EBV in cervical cancer was tested by examining the presence and expression of EBV genes in cervical carcinoma and normal cervical tissue. The EBV genome was present in 55% (17 of 31) of carcinoma samples and in 26% (9/35) of normal samples [45]. A later study used mRNA *in situ* hybridization to detect mRNA of EBV nuclear antigen 2 in tissue samples from patients with invasive uterine cervical cancer and cervical intraepithelial neoplasia [46]. The authors found EBV nuclear antigen-2 (EBNA2) mRNA in these samples; furthermore, they detected expression of EBNA leader protein 2c by using mRNA in situ hybridization and indirect immunofluorescent staining [47]. Another study demonstrated the significance of cervical co-infection with HPV 16 and EBV in uterine cervical cancer patients: all patients carried IgG-anti-VCA and IgG-anti-EBNA serum and more than 30% (of 48 patients) were also infected with HPV 16 [52].

## Kidney cancer

Kidney cancer includes renal cell carcinoma (RCC) and renal pelvis carcinoma, and it also includes Wilms tumor, a type of kidney cancer that usually develops in children under the age of 5. According to the International Agency for Research on Cancer in 2008, the global incidence of kidney cancer was 273,518 cases (2.3% of all cancer cases) and mortality was at 116,368 cases (1.5% of all cancer mortality cases) making RCC 11th most frequent cancer worldwide. Several studies have revealed an association between kidney cancer and viruses. The World Health Organization estimated that 130–170 million people are chronically infected with hepatitis C virus (HCV), a major cause of cirrhosis, liver failure, and hepatocellular carcinoma. A study by Gordon et al. [53] compared incidences of RCC between anti-HCV positive adults and an anti-HCV negative control group, and found that chronic infection with HCV was associated with an increased risk of RCC. In addition, the seroprevalence of HCV in patients with RCC who participated in the cohort study by Gordon et al. was higher than the overall seroprevalence of HCV in the United States (4.3% and 1.6% respectively).

Interestingly, the results of study by Salehipoor et al. [54] suggested an association between high-risk types of HPV and RCC. In this study, nested PCR was used to detect HPV in 49 patients with clinical and histopathological diagnoses of RCC. Subsequently, 14.29% of RCC tissues tested positive for HPV, but no expression of EBV was detected in the control group. All of the detected HPV types were high-risk ones (type 16 in 3 and 18 in 4 patients).

In concordance with the above findings, results from several studies suggested the involvement of EBV in RCC pathogenesis. In a study by Shimakage et al. [55], EBV mRNA was detected in RCC and nephroblastoma by *in situ* hybridization. The expression of EBV was stronger in clear-cell type and papillary RCC than in chromophobe cell type RCC.

Urinary tract infection history was also shown to play a role in kidney cancer development, as Parker et al. [56] reported a positive association of urinary tract infection history with the risk of renal cell carcinoma development. Urinary tract infection history was also shown to play a role in kidney cancer development, as Parker et al. [56] reported a positive association between the risk of RCC development and a history of urinary tract infections in patients.

## Bladder cancer

Urinary bladder cancer can be divided into distinct types, including transitional cell carcinoma (the most common), squamous cell carcinoma and adenocarcinoma. Bladder cancer is the 7th most common cancer among males and 17th most common malignancy in females. In 2008, bladder cancer was the 9th most common cancer globally [57].

Urinary forms of schistosomiasis that is caused by *Schistosoma haematobium* (*S*.*haematobium*) is an infection commonly associated with an increased risk for urinary bladder cancer. An association between schistosomiasis and urinary bladder cancer is particularly evident in Egypt, where schistosomiasis is endemic. The overall prevalence of *S*.*haematobium* infection in Egypt is 37-48% [58]. Urinary bladder cancer is the most common type of cancer in males, and 2nd most common in females, and accounts for nearly 3% of the total cancer incidence in Egypt (compared to the 5-7th most common cancer in males and 7-14th most common cancer in females in schistosoma-free countries) [58].

Studies conducted by the International Agency for Research on Cancer (IARC) [59] revealed that infections with *S*.*haematobium* predisposed patients to bladder cancer, in particular, squamous cell carcinoma. Individuals heavily infected with S.haematobium were more likely to develop bladder cancer, and at a younger age. Bedwani et al. [60] studied the relationship between the clinical history of schistosomiasis and risk for bladder cancer by analyzing data from case–control studies involving 190 bladder cancer patients and 187 control subjects with non-urinary tract conditions. The study showed that schistosomiasis was associated with an increased risk of bladder cancer and accounted for 16% of bladder cancer cases in that Egyptian population.

Urinary bladder cancer was also shown to be associated with viruses. For example, a meta-analysis [61] revealed that infections with high-risk HPV (HR-HPV) types, especially HPV16, might play a significant role in bladder carcinogenesis. Various studies led to the detection of HPV DNA in benign and malignant neoplasms of the bladder [62-65]. Barghi et al. [66] found HPV DNA in 36% of transitional-cell bladder cancer specimens. Moonen et al. [67] detected HPV DNA in 15% of urinary bladder cancer patients, and HR-HPV DNA in 8% of patients (from a total of 107 patients). The number of specimens positive for HR-HPV DNA increased with the clinical progression of the disease, with HR-HPV detected in 0% of Ta, 12.5% of T1, and 18.2% of T2-T4 stage bladder cancers. HPV16 and HPV18 DNA were detected in nearly 50% of urinary bladder dysplasia and carcinoma specimens when examined using *in situ* hybridization [68]. In another study, HPV16 genetic material was found in 40% of transitional bladder cancer specimens [69]. Despite the results of these studies, the IARC concluded that there is “inadequate evidence” to support a role of HPV in bladder carcinogenesis. The human polyomaviruses BKV and JCV establish persistent infections in humans and possess oncogenic and transforming potential in experimental animal models and cell cultures [54]. The presence of BKV and JCV were detected in urothelial carcinomas of the renal pelvis RCCs [69]. However, no definitive association between BKV and human cancers has been established to date [70,71]. Shen et al. [72], detected JCV DNA using nested PCR in 90.1% (30 out of 33) urothelial carcinoma tissue samples and in 100% (5 out of 5) renal cell carcinoma samples. BKV DNA was detected in only one (3%) urothelial carcinoma tissue sample. Geetha et al. [73], detected elevated levels of BKV large T antigen expression in a bladder carcinoma developed in a simultaneous kidney and pancreas transplant recipient. Such levels were not observed in non-neoplastic urothelium. Based on these findings, the authors suggested that BKV virus might function as an etiologic agent in the development of urinary bladder carcinoma.

## Conclusion

A growing body of evidence indicates that some viruses, bacteria and even helminths might increase cancer risk by promoting general inflammation and subsequently affecting key cellular processes. However, the exact mechanisms by which the products of how pathogens promote or predispose cells to neoplastic transformation are unknown. One exception is HPV, which has been shown to induce carcinogenesis in cervical epithelial cells by inactivating tumor suppressor proteins. However, in cases of ovarian, kidney and bladder cancer we can only infer a role for infectious agents based on their associations with these cancers. More in-depth and mechanistic studies are required to understand the molecular mechanisms, by which infectious agents could promote carcinogenesis. Such studies could lead to identification of novel methods of urogenital cancer treatment.

## Abbreviations

OSE: Ovarian surface epithelium; HPV: Human papillomavirus; PID: Pelvic inflammatory disease; TFI: Tubal factor infertility; CMV: Cytomegalovirus; EBV: Epstein-Barr virus; RCC: Renal cell carcinoma; HCV: Hepatitis C virus; IARC: International Agency for Research on Cancer; HR-HPV: High-risk human papillomavirus.

## Competing interests

The authors declare that they have no competing interests.

## Authors’ contributions

KA, NK and IB performed literature research and composed the manuscript. All authors read and approved the final manuscript.
